# Long-acting beta-agonists plus inhaled corticosteroids safety: a systematic review and meta-analysis of non-randomized studies

**DOI:** 10.1186/1465-9921-15-83

**Published:** 2014-07-19

**Authors:** Gimena Hernández, Mónica Avila, Àngels Pont, Olatz Garin, Jordi Alonso, Laurent Laforest, Christopher J Cates, Montserrat Ferrer

**Affiliations:** 1 Health Services Research Group. IMIM (Hospital del Mar Medical Research Institute), Barcelona Biomedical Research Park, office 144. Doctor Aiguader, 88 | 08003, Barcelona, Spain; 2 Universitat Autònoma de Barcelona, Bellaterra, Spain; 3 CIBER en Epidemiología y Salud Pública, CIBERESP, Madrid, Spain; 4 Pompeu Fabra University (UPF), Barcelona, Spain; 5 UCBL Unité de Pharmacoépidémiologie - UMR 5558 CNRS - Université Claude Bernard, Lyon, France; 6 St George’s University of London, Population Health Sciences and Education, London, UK

**Keywords:** Asthma, Long-acting beta-agonists, Inhaled corticosteroids, LABAs, Serious adverse events, Exacerbations

## Abstract

**Background:**

Although several systematic reviews investigated the safety of long-acting beta–agonists (LABAs) in asthma, they mainly addressed randomized clinical trials while evidence from non-randomized studies has been mostly neglected. We aim to assess the risk of serious adverse events in adults and children with asthma treated with LABAs and Inhaled Corticosteroids (ICs), compared to patients treated only with ICs, from published non-randomized studies.

**Methods:**

The protocol registration number was CRD42012003387 (http://www.crd.york.ac.uk/Prospero). Literature search for articles published since 1990 was performed in MEDLINE and EMBASE. Two authors selected studies independently for inclusion and extracted the data. A third reviewer resolved discrepancies. To assess the risk of serious adverse events, meta-analyses were performed calculating odds ratio summary estimators using random effect models when heterogeneity was found, and fixed effect models otherwise.

**Results:**

Of 4,415 candidate articles, 1,759 abstracts were reviewed and 220 articles were fully read. Finally, 19 studies met the inclusion criteria. Most of them were retrospective observational cohorts. Sample sizes varied from 50 to 514,216. The meta-analyses performed (69,939-624,303 participants according to the outcome considered) showed that odds ratio of the LABAs and ICs combined treatment when compared with ICs alone was: 0.88 (95% CI 0.69-1.12) for asthma-related hospitalization; 0.75 (95% CI 0.66-0.84) for asthma-related emergency visits; 1.02 (95% CI 0.94-1.10) for systemic corticosteroids; and 0.95 (95% CI 0.9-1.0) for the combined outcome.

**Conclusions:**

Evidence from observational studies shows that the combined treatment of LABAs and ICs is not associated with a higher risk of serious adverse events, compared to ICs alone. Major gaps identified were prospective design, paediatric population and inclusion of mortality as a primary outcome.

## Background

Long-Acting Beta-Agonists (LABAs) -salmeterol and formoterol- were introduced in the ‘90s when they demonstrated reducing symptoms and use of rescue medication [1]. Concerns about their safety appeared in 1993 when Castle et al. reported a threefold mortality in a randomized clinical trial (RCT) comparing LABAs with SABAs [2]. Post-marketing reports of adverse events showed an increased risk of death and serious asthma events [3]. The Salmeterol Multicenter Asthma Research Trial was stopped in 2003 after an interim analysis showed a fourfold increased mortality amongst patients randomized to salmeterol vs. placebo [4]. Similar concerns about formoterol were raised by a reanalysis of three RCTs. Meta-analyses of RCTs with LABAs as a monotherapy indicated an increased mortality risk [5,6].

Meta-analyses of RCTs examining the safety of LABAs in combination with inhaled corticosteroids (ICs) showed inconsistent results. Most of them found no significant differences in asthma-related hospitalizations and asthma-related mortality compared with patients treated with ICs alone [7-11]. But a statistically significant increase of catastrophic asthma events for LABAs plus ICs was shown by the update of a meta-analysis [12]. In 2010, the Federal Drug Administration (FDA) required label changes to indicate contraindication of use of LABAs without concomitant ICs, recommending only fixed-dose LABAs plus ICs combination, and calling for new studies to address this issue [13].

Nevertheless, there is still a lack of knowledge regarding LABAs’ safety with concomitant ICs use, with both theoretical arguments and limited empirical evidence that ICs may mitigate LABA-associated risks [14-16]. Most of the systematic reviews currently available are based on RCTs, which may present limitations to assess long-term and rare outcomes [5,8-11]. Moreover, RCTs may not reflect the actual patterns of use of these medications in asthma patients’ day-to-day regarding treatment duration and adherence. To our knowledge, there is only one systematic review of observational studies [17]. Its meta-analysis showed that the combined treatment was associated with a lower risk of asthma-related hospitalizations and/or emergency room visits.

Since year 2008, end date of the above mentioned review, many non-randomized studies have been published, especially due to the FDA’s 2010 call for further evidence. The aim of this study was to assess the risk of serious adverse events in patients with asthma treated with LABAs and ICs in comparison to patients treated only with ICs, by synthesizing the available evidence from non-randomized studies through systematic review and meta-analysis.

## Methods

The protocol registration number was CRD42012003387 (http://www.crd.york.ac.uk/Prospero). We searched MEDLINE and EMBASE databases with a specific strategy (see Additional file 1) from 1990, when LABAs were commercialized, to January 20^th^, 2013.

We looked for non-randomized studies in all languages (non-randomized controlled trials, controlled before-after studies, prospective or retrospective cohorts, case-control studies) on adults, adolescents or children with asthma diagnosis. Studies assessing treatment with LABAs plus ICs (either as two separate inhalers or as a single inhaler) compared with ICs monotherapy were considered, regardless of the dose (see Additional file 1). Co-therapy such as immunomodulators and leukotriene modifiers were not excluded. We defined ‘severe exacerbation’ following the American Thoracic Society/European Respiratory Society statement [18] which was based on urgent health care utilization: asthma-related emergency department (ED) visits, hospitalizations, intubations, intensive care unit (ICU) admissions, and use of systemic corticosteroids were considered either specific or combined outcomes.

Two members of the study team, a physician (GH) and a pharmacist (MA), independently reviewed studies found in the literature search by examining titles, abstracts, and full text articles. A third reviewer (MF) resolved discrepancies. A pilot test was performed to homogenize criteria among reviewers. Finally, the selected articles’ reference lists were reviewed to identify other possible studies that could be included.

Data were extracted by agreement of two reviewers using a standardized, predefined data collection form, including: study and participants characteristics, interventions, comparator, outcomes, asthma severity, co-medication, and ethics consideration of each study. Authors were contacted if clarification was needed.

The risk of bias in the identified studies was assessed using a checklist developed by members of the Cochrane Non-Randomised Studies Methods group [19]. We assessed 4 categories of potential biases: groups of comparison, reasons for allocation in groups, parts of the study that were prospective, and group comparability (Additional file 1).

### Analytic strategy

Reported adjusted OR and 95% confidence intervals (95% CI) for the comparison of ICs plus LABAs versus ICs alone were considered. Where adjusted ORs were not reported, unadjusted ORs were held. To assess the risk of severe exacerbation in patients with asthma treated with LABAs plus ICs, compared to those treated only with ICs, meta-analyses were carried out for individual specific adverse events and combined outcomes. Subgroup analyses for children and administration mode were planned. The summary OR and 95% CI estimated in the meta-analyses, together with ORs from individual studies, were presented in forest plots.

Heterogeneity among studies was evaluated using Galbraith plot and I^2^ statistic categorized as follows: <30% not important; 30%-50% moderate; 50%-75% substantial; and 75%-100% considerable [19]. If significant heterogeneity was identified among studies, further examination of the individual studies was conducted, and random effects models (Dersimonian-Laird Method) were used to obtain the summary OR estimates. Otherwise, fixed effects models were used (Mantel-Haentzel Method). Publication bias was assessed by Egger regression asymmetry test and funnel plots. The meta-analytic software program used was STATA.12.

## Results

### Literature search results

**Figure 1:**
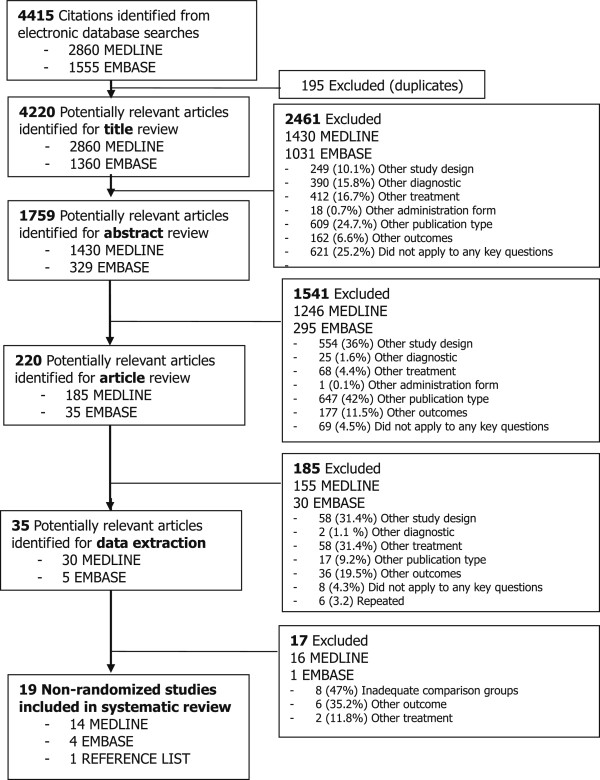
Flow chart diagram.

The literature search identified 4,415 articles (Figure 1). After excluding 195 duplicates, 4,220 titles and 1,759 abstracts were reviewed, reading fully 220 articles. The most frequent reason for exclusion during title and abstract review was “did not apply to any key question” (25.2%), and “other publication type” (42%), respectively; and during full text review, presenting “other study designs” (31.4%) or evaluating “other treatments” (31.4%). Detailed reasons for excluding manuscripts at each step are displayed in Additional file 1. Seventeen of the potentially relevant articles were excluded after full text reading (characteristics are shown in Additional file 1). Finally, 19 studies met the inclusion criteria.

### Characteristics of included studies

Main characteristics of included studies are displayed in Table 1. The majority (16/19) were retrospective observational cohorts based on pharmacy claims from insurance databases. These studies analysed patients with asthma who had initiated an inhaled treatment with LABAs plus ICs or ICs alone. There was also 1 prospective observational cohort, 1 case-control study and 1 before-after study. All the articles described studies carried out in either USA (16/19) or UK (3/19). Regarding sample size, number of participants varied from 50 (Nguyen WT et al. 2005) [20] to 514,216 (Guo JJ et al. 2011) [21]. All articles included have been approved by their Ethics Committee

**Table 1:** Characteristics of included studies

**Author and publication year**	**Study design**	**Sample size (n)**	**Age (years)**	**Administration mode**	**Follow-up period**	**Ascertainment of asthma**	**Outcomes**		**Endpoint measure**
							**Specific**	**Combined**	
Wells et al., 2012 [22]	RC	1,828	12-56	Single inhaler	*2.1(2.0) years	Asthma treatment	-------	1- Asthma-related hospitalization OR asthma-related ED visit OR Systemic Corticosteroid use	aHR
Jacobs et al., 2012 [23]	C-C	181	4-18	Not stated	NA	Clinical diagnosis	1- ICU admission	-------	aOR OR
							2- Deaths		
							3- Intubation		
							4- Positive air pressure use		
Stanford et al., 2012 [24]	RC	10,837	65-79	Single inhaler	12 months	Claims for asthma	1- Asthma-related hospitalization	1- Asthma-related hospitalization OR asthma-related ED visits	aHR
							2- Asthma-related ED visits		
							3- Systemic Corticosteroid use		
Guo et al., 2011 [21]	RC	514,216	0-40	Single & Separate inhalers	-	Claims for asthma	-------	1- Asthma-related hospitalization OR asthma-related ED visits OR Asthma-related intubations	aHR
Stanford et al., 2010 [25]	RC	50,428	> 4	Single inhaler	*290.4 (102.8) days	Claims for asthma	1- Asthma-related hospitalization	1- Asthma-related hospitalization OR asthma-related ED visits	aHR
							2- Asthma-related ED visits		
Hagiwara et al., 2010 [26]	RC	894	12-64	Single inhaler	3-12 months	Claims for asthma	1-Asthma-related hospitalization	1- Hospitalization OR ED visits	aOR
							2-Asthma-related ED visits	2- Hospitalization OR ED visits OR Systemic Corticosteroid use	
							3-Use of SABAs		
Delea et al., 2010 [27]	RC	1,744	> 12	Single inhaler	3-12 months	Claims for asthma	1- ED visits	1- ED visits OR Hospitalization	aOR
								2- ED visits OR hospitalization OR Systemic Corticosteroid use	
de Vries et al., 2010 [28]	RC	467,639	>18	Not stated	5 years	Claims for asthma	1- All mortality;	-------	aRR
							2- Asthma-related mortality		
							3-Asthma-related hospitalization		
							4-GP visits for exacerbation		
Lee et al., 2010 [29]	RC	28,074	18-56	Single & Separate inhalers	12 months	Claims for asthma	1- Asthma-related hospitalization	-------	OR
							2-Asthma-related ED visits		
							3-Systemic Corticosteroid use		
							4- SABAs use		
Thomas et al., 2009 [30]	RC	64,348	10-58	Single & Separate inhalers	12 months	Claims for asthma and asthma treatment	1- Respiratory Hospitalization	1- Asthma-related hospitalization OR asthma-related ED visits OR > 2 prescription of Systemic Corticosteroid uses OR SABA prescription	aOR
							2- Systemic Corticosteroid use		
							3- SABAs use		
Stanford et al., 2008 [31]	RC	58,270	> 12	Single inhaler	12 months	Claim for asthma	1-Asthma-related Hospitalization	1- Asthma-related ED visits OR asthma-related Hospitalization	aOR
							2- Asthma-related ED visits		aHR
Campbell et al., 2008 [32]	PC	684	> 18	Single inhaler	24 months	Severe asthma	-------	1- Asthma-related hospitalization OR asthma-related ED visit OR Systemic Corticosteroid use	aOR OR
Colice et al., 2008 [33]	RC	1,283	6-64	Not stated	12 months	Claims for asthma	1- Asthma-related hospitalization	-------	OR
							2- Asthma-related ED visits		
Delea et al., 2008 [34]	RC	2,269	> 5	Single & Separate inhalers	12 months	Claims for asthma	1- Asthma-related hospitalization	1- Asthma-related hospitalization OR ED visits OR Systemic Corticosteroid use OR alternative study medication	aOR
							2- Asthma-related ED visits	2- Asthma-related hospitalizations OR ED visits OR oral corticosteroid	
							3- Oral corticosteroids use	3- Asthma-related hospitalization OR ED visits hospitalization	
Friedman et al., 2007 [35]	RC	5,503	12-65	Single inhaler	12 months	Claims for asthma	1-Asthma-related hospitalization	-------	aOR
							2-Asthma-related ED visits		
							3- Any ED visits		
Zhang et al., 2007 [36]	RC	2,596	15-55	Single & Separate inhalers	12 months	Claims for asthma	1- Oral corticosteroid use	1- Asthma-related hospitalization OR asthma-related ED visits	OR
							2- SABA use		
Stempel et al., 2006 [37]	RC	9,192	4-17	Single inhaler	12 months	Claims for asthma	1- SABA use	1- Asthma-related hospitalization OR asthma-related ED visit	aRR
							2-Corticosteroids use		
O’Connor et al., 2005 [38]	RC	2,414	> 15	Single & Separate inhalers	12 months	Claims for asthma	-------	1- Asthma-related hospitalization OR ED visits	aOR aHR
Nguyen et al., 2005 [20]	B-A	50	4-17	Single inhaler	12 months	Enrolled patients	1- Asthma-related hospitalization	-------	aRR
							2- Asthma-related ED visits		

*RC*: Retrospective Cohort; *C-C*: Case-control study; *PC*: Prospective cohort; *B-A*: Before-after study; * = Mean (SD); *SABA* = Short- Acting Beta-Agonist; *ED* = Emergency Department; *HR* = Hazard Ratio; *OR* = Odds Ratio; *aHR* = Adjusted HR; *aOR* = Adjusted OR.

### Assessment of risk of bias in individual studies

**Figure 2:**
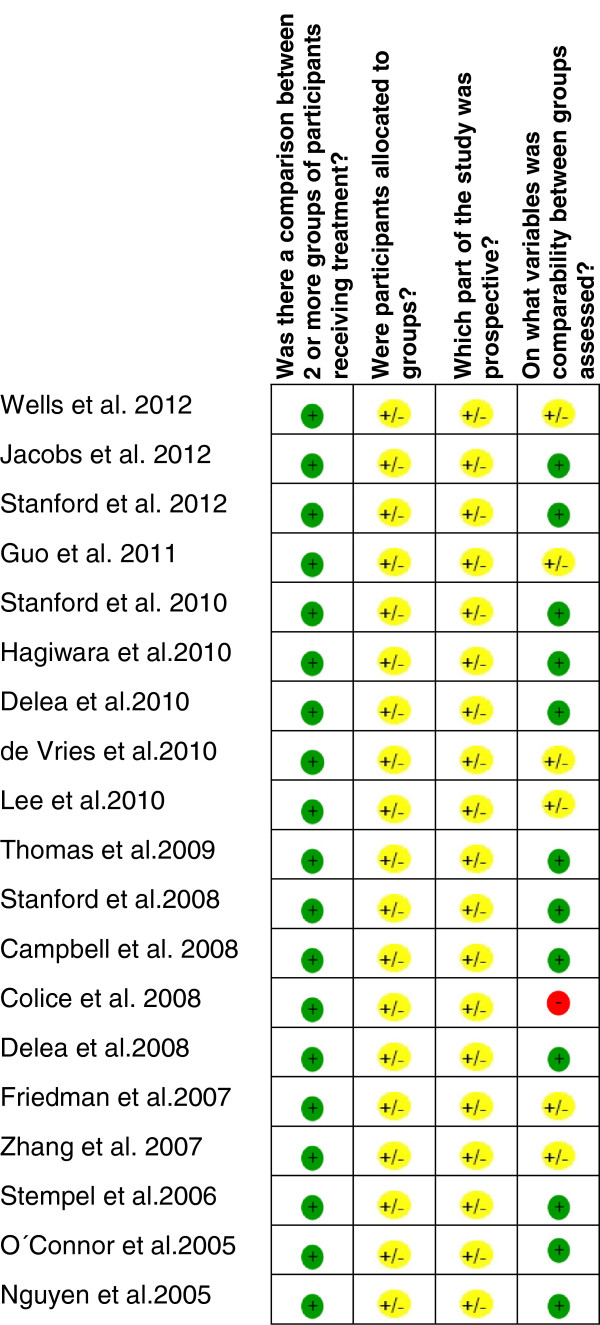
Risk of bias assessment in individual studies.

An overview of the risk of bias in individual studies is shown in Figure 2. First, all studies compared the LABAs plus ICs group with the ICs alone group, as this was an inclusion criterion. Therefore, risk of bias in this item was not identified. Second, risk related to allocation was intermediate since patients were allocated by treatment decisions and not by location differences, participant’s preferences, or based on outcomes. Third, we considered the risk related to retrospective design as intermediate, because the outcomes assessment was retrospective and the generation of hypothesis was prospective. Fourth, risk of bias related to groups’ comparability was not identified in most cases because ORs were adjusted for potential confounders and studies compared outcome variables at baseline. Only the study of Colice et al. [33] had not done either of these two procedures (red mark on Figure 2).

### Meta-analyses results

Of the 19 studies identified, 6 were not included in the meta-analyses performed (4 retrospective cohorts, the case-control, and the before-after study) because they did not provide any of the specific estimators assessed. The most commonly reported outcomes were emergency department (ED) visit and asthma-related hospitalization (reported in 9 and 8 studies, respectively), followed by systemic corticosteroid use (4 studies). There were also two commonly combined outcomes: asthma-related hospitalizations, asthma-related ED visits or systemic corticosteroid (5 studies); and asthma-related hospitalizations or asthma-related ED visits (9 studies). The latter meta-analysis was not reported because it presented considerable heterogeneity (I^2^ = 93%).

Subgroup analyses concerning age and administration mode (single or separate inhalers) could not be performed due to the lack of studies providing disaggregated information for these groups. The three studies focused on children and adolescents had different designs (case-control, before-after and retrospective), and only two of the four retrospective cohorts which included adults and children stratified their analysis by age subgroups. Regarding administration mode, 10 studies included only users of fixed-dose LABAs plus ICs in a single inhaler, three studies did not provide this information, and only three of the six studies which included LABAs plus ICs both as single or two separate inhalers performed disaggregate analysis (Guo et al. [21], Delea et al. [34], and O’Connor et al. [38]).

#### Asthma-related hospitalizations

**Figure 3:**
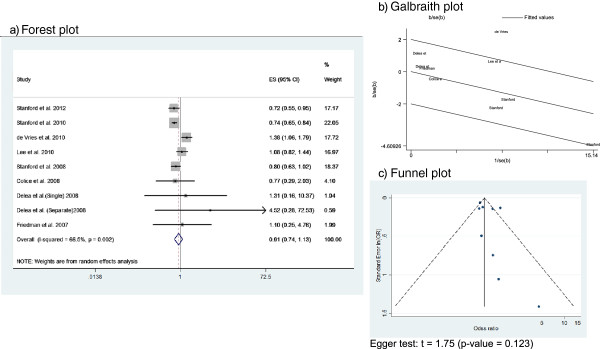
This figure includes Forest plot (a), Galbraith plot (b), and Funnel plot (c) of the asthma-related hospitalization meta-analysis.

Figure 3 shows the Forest plot (Figure 3a), Galbraith plot (Figure 3b), and Funnel plot (Figure 3c) of the asthma-related hospitalization meta-analysis. Estimators of this outcome were provided by 8 of the retrospective cohorts. Overall, these studies included 624,303 patients. Results from the study by Delea et al. [34] were included as 2 different estimators because specific ORs for single and separate inhalers (instead of an overall OR) were provided. The ORs of the individual studies ranged from 0.72 (95% CI 0.55-0.95) reported by Stanford et al. [24] to 4.52 (95% CI 0.28-72.53) reported by Delea et al. [34]. The summary OR was 0.88 (95% CI 0.69-1.12). Random effect models were used due to substantial heterogeneity (I^2^ = 66%). The Galbraith plot (Figure 3b) showed that all points except the study corresponding to deVries et al. [28] fell within the confidence limits. However, this has a considerable weight due to the large sample size (n = 467,639). The Funnel plot (Figure 3c) seems symmetric and Egger’s test was non-significant, which suggests that there was no publication bias.

#### Asthma-related ED visits

**Figure 4:**
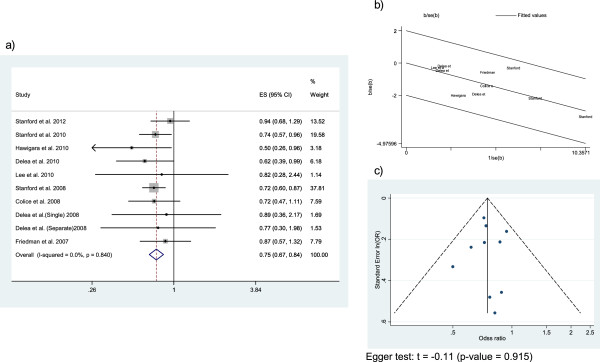
This figure includes Forest plot (a), Galbraith plot (b), and Funnel plot (c) of the asthma-related emergency department visits.

The forest plot of the risk of asthma-related ED visits was constructed from 9 studies including 153,799 patients (Figure 4). All the ORs of the individual studies were lower than 1 and the overall summary OR was 0.75 (95% CI 0.66-0.84). A fixed effect model was used because there was no heterogeneity. Galbraith plot showed that most studies fell within the confidence limits, and the Funnel plot suggested no publication bias.

#### Asthma-related systemic corticosteroid use

**Figure 5:**
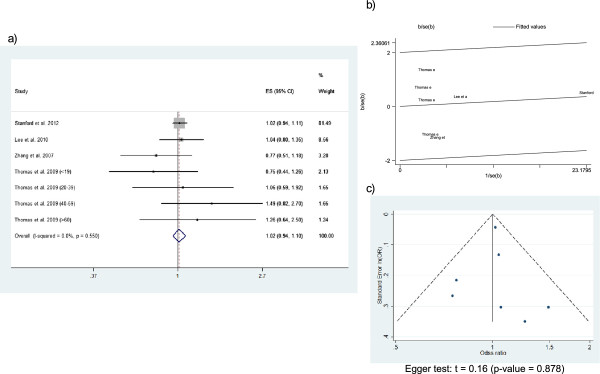
This figure includes Forest plot (a), Galbraith plot (b), and Funnel plot (c) of the asthma-related systemic corticosteroid use.

Four studies (105,855 patients in total) provided estimators of asthma-related systemic corticosteroid use risk (Figure 5). Results from the study by Thomas et al. [30] were included as four separate estimators because ORs were provided for each age group. The summary OR was 1.02 (95% CI 0.94-1.10), calculated with a fixed effects model as no heterogeneity was found. All studies fell inside the confidence limits of Galbraith plot, and the funnel plot appeared symmetric.

#### Combined outcome of asthma-related hospitalizations, asthma-related ED visits or systemic corticosteroid use

**Figure 6:**
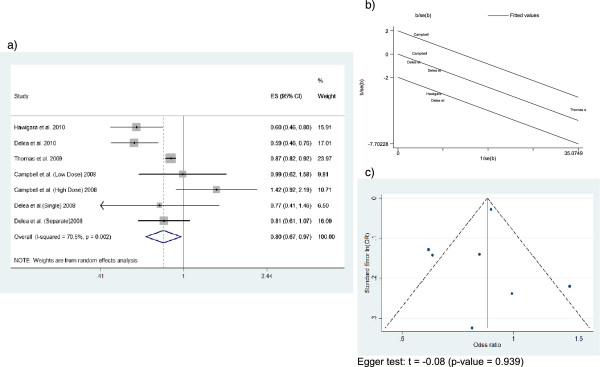
This figure includes Forest plot (a), Galbraith plot (b), and Funnel plot (c) of the asthma-related hospitalization or emergency department visits or systemic corticosteroid use.

Data from 5 studies were available for severe asthma exacerbations meta-analysis (Figure 6), defined as asthma-related hospitalizations, asthma-related ED visits or systemic corticosteroid use. Overall, these studies included 69,939 patients and the summary OR was 0.95 (95% CI 0.9-1). Results from the study by Campbell et al. [32] were included as two separate estimators because ORs were provided for both low and high corticosteroid doses. The latter is the only individual estimator above 1 (OR = 1.42; 95% CI 0.92-2.19). A random effects model was used, as substantial heterogeneity was found (I^2^ = 70%). Figure 6b shows that estimators provided by Campbell et al. [32], Hagiwara et al. [26], and Delea et al. [34] fell just outside the confidence limits. Similarly, three estimators are placed outside the triangle in the funnel plot. As there are only 5 studies included in this meta-analyses, Egger’s test cannot be interpreted.

## Discussion

To date, less than 10% of all systematic reviews have adverse events’ assessment as a primary objective [39]. Our findings support the relevance and suitability of performing systematic reviews of harms to provide valuable information on these risks. This systematic review identified 19 studies which met the inclusion criteria: 16 retrospective cohorts, 1 prospective cohort, 1 case-control, and 1 before-after study (1,165,342 participants). The meta-analyses performed (69,939-624,303 participants according to the outcome considered) showed that the LABAs/ICs combined treatment was not associated to a higher risk of adverse events, when compared with ICs alone. The OR ranged from 0.75 to 1.02 for the different outcomes explored, which is congruent with findings from meta-analyses of RCTs assessing asthma-related serious adverse events for salmeterol (0.95; 95% CI 0.52-1.73) [8] and formoterol (0.53; 95% CI 0.28-1.0) [10].

This consistency between our results and those from meta-analyses of RCTs reinforces the evidence available on this topic. It is well known that RCTs are the gold standard in evaluating efficacy and safety of emerging therapies. However, their poor external validity [40] is a particular concern for long term chronic conditions that affect large and heterogeneous patient populations, such as asthmatics. In fact, it has been estimated that only 1.2% [41] or 5% [42] of the usual care asthma population could have been eligible for a typical asthma RCT. In this context, despite potential issues regarding observational studies’ internal validity, they are gaining widespread recognition [43,44] providing valuable information on treatment effectiveness and safety, especially in long-term outcomes.

To our knowledge, this is the first systematic review of non-randomized studies including children and adults with asthma to assess adverse events of LABAs, as the only systematic review of observational studies previously published was limited to adult patients (asthma-related hospitalizations OR = 0.85; 96%CI 0.74-0.97) [17]. It included mainly unpublished studies identified from a pharmaceutical company’s research register. Since then, the publication of 12 observational studies permitted the inclusion of a larger number of patients. In comparison with systematic reviews of salmeterol and formoterol RCTs (with 15,309 and 13,366 patients, respectively), synthesis of non-randomized studies provides results from larger representative asthma samples, more accurate reflection of the usual clinical practice, and longer follow-up periods. The follow-up periods of studies included in our systematic review ranged from 3 months to 5 years, being in most cases 1 year (12 studies), an adequate frame of time for the assessment of adverse events [19].

We identified several limitations on our review process. First, four retrospective cohorts could not be included in any of the meta-analyses performed due mainly to the lack of the specific estimator needed, but their results were consistent with our findings [21,22,37,38]. Second, outcomes of these retrospective cohorts varied substantially, from systemic corticosteroids use to deaths. Related to this wide range of clinical outcomes, there was a limitation for synthesizing them by meta-analysis – mortality, a primary outcome of interest, was reported only by one study [28]. Furthermore, the use of composite endpoints could give misleading conclusions because the components have different relevance [45]. However, not only the composite endpoints, but also the individual adverse events which compose them have been considered in the meta-analyses. Third, internal validity of the summary provided by a meta-analysis depends on the quality of primary studies. Confounding and selection bias could distort the findings from observational studies and therefore meta-analyses including them would produce biased estimates also. In our systematic review, sensitivity analysis by quality assessment was not performed as risk of bias was homogeneous among studies. Quality assessment was considered moderate for most of them because the studies were mainly comparative, allocation was based on treatment decisions, and adjusted by potential confounders. In fact, only few unadjusted estimators were included in the meta-analysis, and the sensitivity analyses carried out to assess the impact of excluding them, showed similar summary ORs: 0.89 (95% CI 0.69-1.15) for asthma-related hospitalization and 0.75 (95% CI 0.67-0.85) for asthma-related ED visits.

Most of the retrospective cohort studies identified in this systematic review obtained data from administrative medical claims and electronic health records, with definitions based on medication prescriptions and ICD-9 diagnosis (i.e. asthma codes for inclusion and other respiratory conditions for exclusion). The main limitations derived from designs of this nature include: a) presence of a prescription claim does not necessarily indicate that the medication was taken; and b) asthma severity criteria were not applied in most studies, and in those that did, severity definitions were based on medication use instead of spirometry or clinical parameters. To balance treatment groups, most of the studies made adjustments on baseline risk factors and socioeconomic variables by using regression models and propensity score matching. Nevertheless, possible confounding factors such as severity and adherence could still remain.

The planned subgroup analysis for children and administration mode (as single or two separate inhalers) could not be conducted, and merits further comments. Stanford et al. [25] performed an analysis stratified by age groups with similar results for adults and children aged 4-18 years: OR was 0.917 (95% CI 0.85-0.98) for ED visit and 0.88 (95% CI 0.7-1.11) for hospitalization. The case-control study by Jacobs et al. [23] showed that paediatric LABA use in combination with ICs did not increase the likelihood of intensive care unit admission among hospitalized children, compared to ICs alone. Regarding administration mode, the little available evidence is controversial. The largest retrospective cohort identified in this review [21] is remarkable because it showed higher risk for single inhalers compared with separate inhalers on a combined outcome composed of asthma-related hospitalizations, intubations or asthma-related ED visits: OR of 1.13 (95% CI 1.09-1.16) among newly diagnosed patients, and OR of 1.12 (95% CI 1.10-1.12) among those with pre-existing asthma. On the contrary, in the study by O’Connor et al. [38] patients receiving LABAs plus ICs in a single inhaler were less likely to have an ED visit or to be hospitalized, compared with patients receiving the same treatment in separate inhalers (OR 0.69, 95% CI 0.51-.95). Delea et al. [34] showed similar results in both administration modes.

Heterogeneity was substantial (66.5% and 70.5%) in two of the four meta-analyses reported. In those conducted with asthma-related hospitalization risk, the only study out of the confidence limits in the Galbraith plot was deVries et al. [28]. This study differs from the other ones in having a follow-up period of 5 years, but many other possible reasons could explain such heterogeneity. In the meta-analysis conducted with the combined outcome, the only estimator that fell outside the Galbraith plot limits was the group with high dose of corticosteroids and salmeterol in Campbell et al. [32], with an OR higher than 1. This might reflect that despite the adjustments, patients taking high corticosteroid doses represented a more severe group.

Almost two thirds of the studies were performed by Glaxo Smith Kline Beecham, while others received industry support without describing the extent of involvement of their sponsors. Usually, publication bias refers to the journals’ rejection of studies with negative results. Yet safety studies sponsored by the pharmaceutical industry could suffer from publication bias in the opposite direction, as it is more likely to publish negative results and to select the most favourable outcomes. We have found no evidence of publication bias in the meta-analyses reported, but Egger’s test has limited power when the number of studies is low, and funnel plots may have subjective interpretation.

## Conclusions

The current evidence from non-randomized studies shows that combined treatment of LABAs and ICs is not associated with higher risk of serious adverse events. Our systematic review identified major gaps in the available literature; accordingly our key recommendations for further research are to conduct prospective cohort studies, to perform studies among the paediatric population, and to include mortality as a primary outcome. Accumulative valid data is needed to allow evidence-based decisions taking into account safety of LABAs plus ICs in asthma treatment.

## Abbreviations

ICs: Inhaled corticosteroids; LABAs: Long-acting beta2–agonists; SABAs: Short acting beta agonists; RCT: Randomized clinical trial; FDA: Federal Drug Administration; ED: Emergency department; ICU: Intensive care unit admissions; CI: Confidence intervals; HR: Hazard ratio.

## Competing interests

The authors declare that they have no competing interests.

## Authors’ contributions

GH contributed to the conception and design of the article, conceptualized and oversaw analyses, contributed to the interpretation of data, and wrote the article. MA contributed to the reviewing and web search of included and excluded articles. AP contributed to the analysis and gave statistical support. OG, JA, CC, LL oversaw all aspects and reviewed the article for important intellectual content. MF oversaw all aspects, contributed to the conception and design of the article, contributed to the statistical analyses, carried out the interpretation of data, and contributed to the writing of the article. All the co-authors critically revised the manuscript and approved the final draft before submission.

## Supplementary Material

Additional file 1 The additional file contains the search strategy, the inclusion and exclusion criteria, the risk of bias assessment tool and information regarding the articles excluded at each step of the Systematic Review. Click here for file
