# Supplementary material for: Long-acting beta-agonists plus inhaled corticosteroids safety: a systematic review and meta-analysis of non-randomized studies
Source: Respir Res. 2014 Jul 19;15(1):83. doi: 10.1186/1465-9921-15-83 (PMC4132190; doi:10.1186/1465-9921-15-83)
Supplement: Additional file 1 — The additional file contains the search strategy, the inclusion and exclusion criteria, the risk of bias assessment tool and information regarding the articles excluded at each step of the Systematic Review. [file 1465-9921-15-83-S1.pdf]

<u>**Online Supplementary Material**</u>

LABAs plus Inhaled Corticosteroids safety: a systematic review of non-randomized studies

Hernández G, Avila M, Pont A, O.Garin, Laforest L, Cates C, Alonso J, Ferrer M, and the ASTROLAB group.

eMethods

I. Search Strategy

eTables

I. Study Inclusion and Exclusion criteria
II. Quality assessment of each article
III. Reasons for exclusion in each step of the Systematic Review
IV. Characteristics of excluded studies

eMethods1 . Search Strategy

MEDLINE

(((asthma[MeSH Terms]) OR asthma*[Text Word]) AND (((((adrenergic beta 2 agonists[MeSH Terms]) OR long acting*[Text Word]) OR long-acting*[Text Word]) OR LABA*[Text Word]) OR salmeterol[Text Word]) OR formoterol[Text Word]) NOT clinical trials, randomized[MeSH Terms].

EMBASE

('asthma'/exp OR asthma AND [humans]/lim AND [2000-2012]/py) AND ('beta 2 adrenergic receptor stimulating agent'/exp OR 'formoterol'/exp OR 'salmeterol'/exp OR (laba AND [2000-2012]/py))

eTable I. Study Inclusion and Exclusion Criteria

PICO criteria	Inclusion and exclusion criteria
**Population and condition of interest**	Study design included in one of these: non-randomized controlled trials, controlled before-after studies; prospective cohort studies, retrospective cohort studies, historically controlled trials, case-control studies and nested case-control studies.<br>Studies that enrolled patients with asthma diagnosed.<br>Studies included adults, children and mixed age population (adult and children)
**Interventions**	All studies included LABAs combined with ICs (single or separate inhalers)
**Comparator**	ICs alone (co-medication was admitted such as SABA, inmunomodulators)
**Outcomes**	We included studies that reported the following outcomes: severe exacerbation (use of systemic corticosteroids, asthma-related emergency room visits, hospitalizations, intubations, or intensive care unit admissions), moderate exacerbation (use of SABA) , adverse events, death

eTableII: Risk of bias assessment Cochrane´s Checklist

**Item**
**Was there a comparison between 2 or more groups of participants receiving different interventions?**
**Were participants allocated to groups:**
- by treatment decisions?
- by participant's preferences?
- on the basis of outcome?
**Which part of the study was prospective?**
- Identification of participants*
- Assessment of baseline and allocation to intervention?*
- Assessment of outcomes?
- Assessment of hypothesis?
**On what variables was comparability between groups assessed?**
- Potential confounders?
- Baseline assessment of outcome variables?

only for Prospective Studies

Options: YES / No / Possibly

eTable III: Reasons for exclusion in each step of the Systematic Review

MEDLINE	Review of titles	Review of abstracts	Review of manuscripts
**1. Inclusion (eligible for next review step)**			
**2. Study design**			
2a. Randomized clinical trial	116 (8.1%)	429 (34.4%)	22 (14.2%)
2b. Ecological	1 (0.1%)	2 (0.2%)	1 (0.6%)
2c. Health economic evaluations	37 (2.6%)	20 (1.6%)	1 (0.6%)
2d. Systematic Review / Meta-analysis	51 (3.6%)	30 (2.4%)	
2e. Pooled		2 (0.2%)	2 (1.3%)
2x. Others	7 (0.5%)	27 (2.2%)	31 (20%)
**3. Population**			
3a. COPD	79 (5.5%)	4 (0.3%)	1 (0.6%)
3X. Others	52 (3.6%)	15 (1.2%)	3 (0.6%)
**4. Treatment**			
4a. SABAs	20 (1.4%)	3 (0.2%)	3 (1.9%)
4b. Omalizumab / Lebrikizumab	34 (2.4%)	3 (0.2%)	1 (0.6%)
4c. Antileukotriens: Montelukast/Pranlukast	51 (3.6%)	2 (0.2%)	5 (3.2%)
4d.Bronchial termoplasty		2 (0.2%)	
4X. Others	91 (6.4%)	14 (1.1%)	46 (29.7%)
**5. Administration Form**			
5a. Oral	7 (0.5%)	1 (0.1%)	
5X. Others	4 (0.3%)		
**6. Publication type**			
6a. Reviews	238 (16.6%)	546 (43.7%)	2 (1.3%)

6b. Case series / case report	22 (1.5%)	8 (0.6%)	2 (1.3%)
6c. Letter to the editor	22 (1.5%)	1 (0.1%)	
6d. Comments	71 (5.0%)	18 (1.4%)	
6e. Guidelines	29 (2.0%)	1 (0.1%)	
6X. Others	8 (0.6%)	4 (0.3%)	
**7. Outcome**			
7c. HRQL and other PRO	14 (1.0%)	14 (1.1%)	2 (1.3%)
7X. Others	50 (3.5%)	50 (4.0%)	29 (18.7%)
**8. Others**			
8a. Pathophisiology and medication	257 (18.0%)	18 (1.4%)	3 (1.9%)
8b. No thumans	11 (0.8%)	2 (0.2%)	3 (1.9%)
8c. Genetics	71 (5.0%)	1 (0.1%)	
8d. Diagnostic	59 (4.1%)		
8X. Others	28 (2.0%)	29 (2.3%)	
**TOTAL**	1430	1246	155

EMBASE	Review of titles	Review of abstracts	Review of manuscripts
**1. Inclusion (eligible for next review step)**			
**2. Study design**			
2a. Randomized clinical trial	12 (1.2%)	10 (3.4%)	
2b. Ecological		2 (0.7%)	
2c. Health economic evaluations	14 (1.4%)	11 (3.7%)	
2d. Systematic Review / Meta-analysis	10 (0.1%)	3 (1%)	
2e. Pooled			
2x. Others	1 (0.01%)	18 (6.1%)	1 (3.3%)
**3. Population**			
3a. COPD	50 (4.8%)	3 (1%)	
3b. Rhinitis	6 (0.6%)		
3c.Allergy	15 (1.5%)	1 (0.3%)	
3X. Others	188 (18.2%)	1 (0.3%)	
**4. Treatment**			
4a. SABAs	9 (0.9%)	5 (1.7%)	
4b. Omalizumab / Lebrikizumab	44 (4.3%)	4 (1.4%)	1 (3.3%)
4c. Antileukotriens: Montelukast / Pranlukast	55 (5.3%)	4 (1.4%)	
4d.Bronchial termoplasty	5 (0.5%)		
4e.Anticholigernics	1 (0.1%)		
4X. Others	102 (9.9%)	31 (10.5%)	2 (6.7%)
**5. Administration Form**			
5a. Oral	1 (0.1%)		
5b. IV	1 (0.1%)		

5X. Others	5 (0.5%)		
**6. Publication type**			
6a. Reviews	33 (3.2%)	56 (19%)	
6b. Case series / case report	135 (13.1%)	12 (4.1%)	
6c. Letter to the editor	6 (0.6%)		
6d. Comments	19 (1.8%)	1 (0.3%)	
6e. Guidelines	18 (1.7%)		
6X. Others	8 (0.8%)		13 (43.3%)
**7. Outcome**			
7a. Congenital malformation	8 (0.8%)		
7c. HRQL and other PRO	17 (1.6%)	4 (1.4%)	
7X. Others	73 (7.1%)	109 (37.1%)	5 (16.7%)
**8. Others**			
8a. Pathophisiology and medication	31 (3%)	2 (0.7%)	
8b. Not humans	1 (0.1%)		
8c. Genetics	65 (6.3%)	2 (0.7%)	
8d. Diagnostic	40 (3.9%)		
8X. Others	58 (5.6%)	15 (5.1%)	2 (6.7%)
**9. Repeated**			6 (20%)
**TOTAL**	1031	294	30

eTableIV: Characteristics of excluded studies

Author	Study characteristics			Type of inhaler	Reason for exclusion	Outcomes
	Study type	Population (N)	Age (Range)			
Elkout et al.2012	RC	3172	0-18	Sin & Sep	Other outcome	Number of SABA and courses of SysCort prescribed annually.
Navaratnam et al.2011	RC	16063	12-65	Single	No LABA in intervention group	Asthma control outcomes
Butland et al. 2011	C-C	1064	1-64	Not stated	Exposure not divided in adequate groups	Asthma deaths
Liao et al.2010	PC	2236	12-54	Not stated	Other outcome	Risk of admission among ED patients with Acute Asthma
Elkout et al. 2010	RC	10454	0-18	Sin & Sep	Comparison IC/LABA vs IC+LABA	Number of SABA prescription, prescription of systemic corticosteroid use
Zhang et al.2009	RC	507966	> 18	Not stated	Other outcome	Acute myocardial infarction
Smith et al. 2009	RC	3013	5-62	Sin & Sep	Outcome not related to medication	Exacerbation requiring hospital visit
Plaza et al.2009	RC	1153	15-69	Not stated	Descriptive analysis of clinical characteristics of asthma exacerbations	Clinical characteristics: mortality, length of stay, gas exchange, lung function.
Navaratnam et al.2009	RC	8188	12-65	Single	No endpoint measure	Mean of exacerbations
Spahn et al.2009	RC	46774	4-55	Single	Comparison group not stated	Asthma-related ED visits and hospitalization

Wang et al.2008	C-C	1940	> 5	Not stated	Exposure not divided in adequate groups	Asthma-related hospitalization
Borderias et al.2007	RC	696	15-55	Not stated	Addition of Montelukast to IC plus LABA treatment	Asthma attacks, use of SABAs
Lyell et al.2005	CC	105	<16	Not stated	Exposure not divided in adequate groups	Factors that may predict severe asthma attacks
Laforest et al.2005	RC	1038	17-50	Not stated	Other outcome	Antibiotic prescription
Angus et al. 2005	RC	588	4-55	Sin & Sep	Comparison IC/LABA vs IC+LABA	SABA use, Oral corticosteroids use
Anderson et al. 2005	C-C	1064	< 65	Not stated	Exposure not divided in adequate groups	Asthma deaths
Chan et al. 2004	RC	2246	12-55	Sin & Sep	Comparison IC/LABA vs IC+LABA	Asthma-related ED visits, asthma-related hospitalization, use of SABA, systemic corticosteroid use

RC: Retrospective Cohort; **C-C:** case-control study; **PC:** Prospective cohort; **BS:** Before-after study; **Sing & Sep**: Single and separate inhalers; **HR**= Hazard Ratio; **OR=**Odds Ratio; **a=**adjusted; **u=**unadjusted; ED = Emergency Room; **PS**= Propensity Score
